# Triethyl­ammonium bis­{2-[(2-oxido-5-nitro­benzylidene)amino]­benzoato}ferrate(III) monohydrate

**DOI:** 10.1107/S1600536811011196

**Published:** 2011-04-13

**Authors:** Eduard N. Chygorin, Svetlana R. Petrusenko, Volodymyr N. Kokozay, Yuri O. Smal, Volodymyr V. Bon

**Affiliations:** a Department of Inorganic Chemistry, National Taras Shevchenko University, 64 Volodymyr’ska St, 01601 Kyiv, Ukraine; b Department of Chemistry of Complex Compounds, V.I. Vernadsky Institute of General and Inorganic Chemistry, National Academy of Sciences of Ukraine, 32/34 Palladin Ave, Kyiv 03680, Ukraine

## Abstract

In the title compound, [NH(C_2_H_5_)_3_][Fe(C_14_H_8_N_2_O_5_)_2_]·H_2_O, the iron(III) ion is hexa­coordinated by four O atoms in the basal plane [Fe—O distances in the range 1.904 (4)–1.909 (4) Å] and two N atoms in the axial plane [Fe—N = 1.981 (4) and 1.985 (4) Å] of two tridentate fully deprotonated 2-{[(2-oxido-5-nitro­phen­yl)methyl­ene]amino}­benzoato (H_2_ *L*) ligands, forming a tetra­gonally elongated octa­hedral geometry. The triethyl­ammonium cations and complex anions are linked by N—H⋯O hydrogen bonds into chains parallel to [100]. Disordered water mol­ecules (occupancy ratio 0.6:0.4) occupy the voids in the crystal structure.

## Related literature

For the stuctures of related compexes, including those with phenyl-salicyliden-imine (PSI) ligands similar to H_2_ *L*, see: Rotondo *et al.* (2009 ▶); Patel (2009 ▶); Patel *et al.* (2008 ▶); Laye & Sanudo (2009 ▶); Lu *et al.* (2006 ▶); Rosair *et al.* (2002 ▶). For bond-valence sums, see: Brown & Altermatt (1985 ▶).
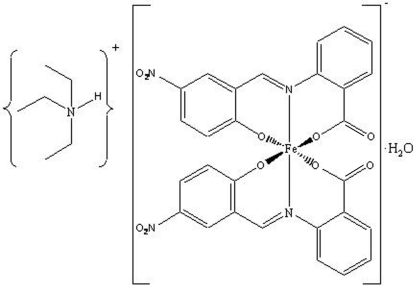


## Experimental

### 

#### Crystal data

(C_6_H_16_N)[Fe(C_14_H_8_N_2_O_5_)_2_]·H_2_O
*M* *_r_* = 742.50
Monoclinic, 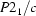

*a* = 10.2688 (3) Å
*b* = 14.7128 (4) Å
*c* = 25.0800 (7) Å
β = 97.230 (2)°
*V* = 3759.03 (18) Å^3^
*Z* = 4
Mo *K*α radiation
μ = 0.46 mm^−1^
*T* = 296 K
0.35 × 0.05 × 0.04 mm

#### Data collection

Bruker APEXII CCD diffractometer
Absorption correction: multi-scan (*SADABS*; Bruker, 2005 ▶) *T*_min_ = 0.855, *T*_max_ = 0.982
20469 measured reflections
6432 independent reflections
5305 reflections with *I* > 2σ(*I*)
*R*_int_ = 0.035

#### Refinement

*R*[*F* ^2^ > 2σ(*F* ^2^)] = 0.081
*wR*(*F* ^2^) = 0.242
*S* = 1.13
6432 reflections
466 parameters
H-atom parameters constrained
Δρ_max_ = 0.89 e Å^−3^
Δρ_min_ = −0.61 e Å^−3^

### 

Data collection: *APEX2* (Bruker, 2005 ▶); cell refinement: *SAINT* (Bruker, 2005 ▶); data reduction: *SAINT*; program(s) used to solve structure: *SHELXS97* (Sheldrick, 2008 ▶); program(s) used to refine structure: *SHELXL97* (Sheldrick, 2008 ▶); molecular graphics: *PLATON* (Spek, 2009 ▶); software used to prepare material for publication: *publCIF* (Westrip, 2010 ▶).

## Supplementary Material

Crystal structure: contains datablocks I, global. DOI: 10.1107/S1600536811011196/zk2002sup1.cif

Structure factors: contains datablocks I. DOI: 10.1107/S1600536811011196/zk2002Isup2.hkl

Additional supplementary materials:  crystallographic information; 3D view; checkCIF report

## Figures and Tables

**Table 1:** Hydrogen-bond geometry (Å, °)

*D*—H⋯*A*	*D*—H	H⋯*A*	*D*⋯*A*	*D*—H⋯*A*
N5—H5*A*⋯O7	0.91	2.00	2.865 (13)	159

**Fig. 1.**
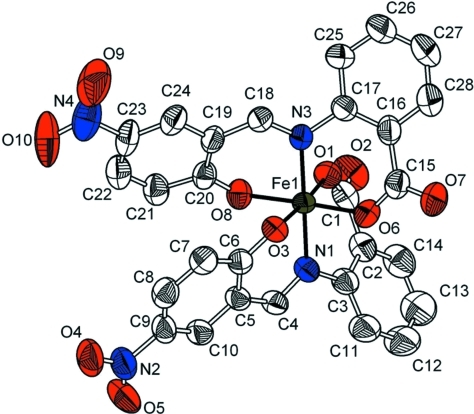
The structure of [Fe(C14H8NO5)2]- with atom labels and 50% probability displacement elipsoides. The H atoms have been omitted.

**Fig. 2.**
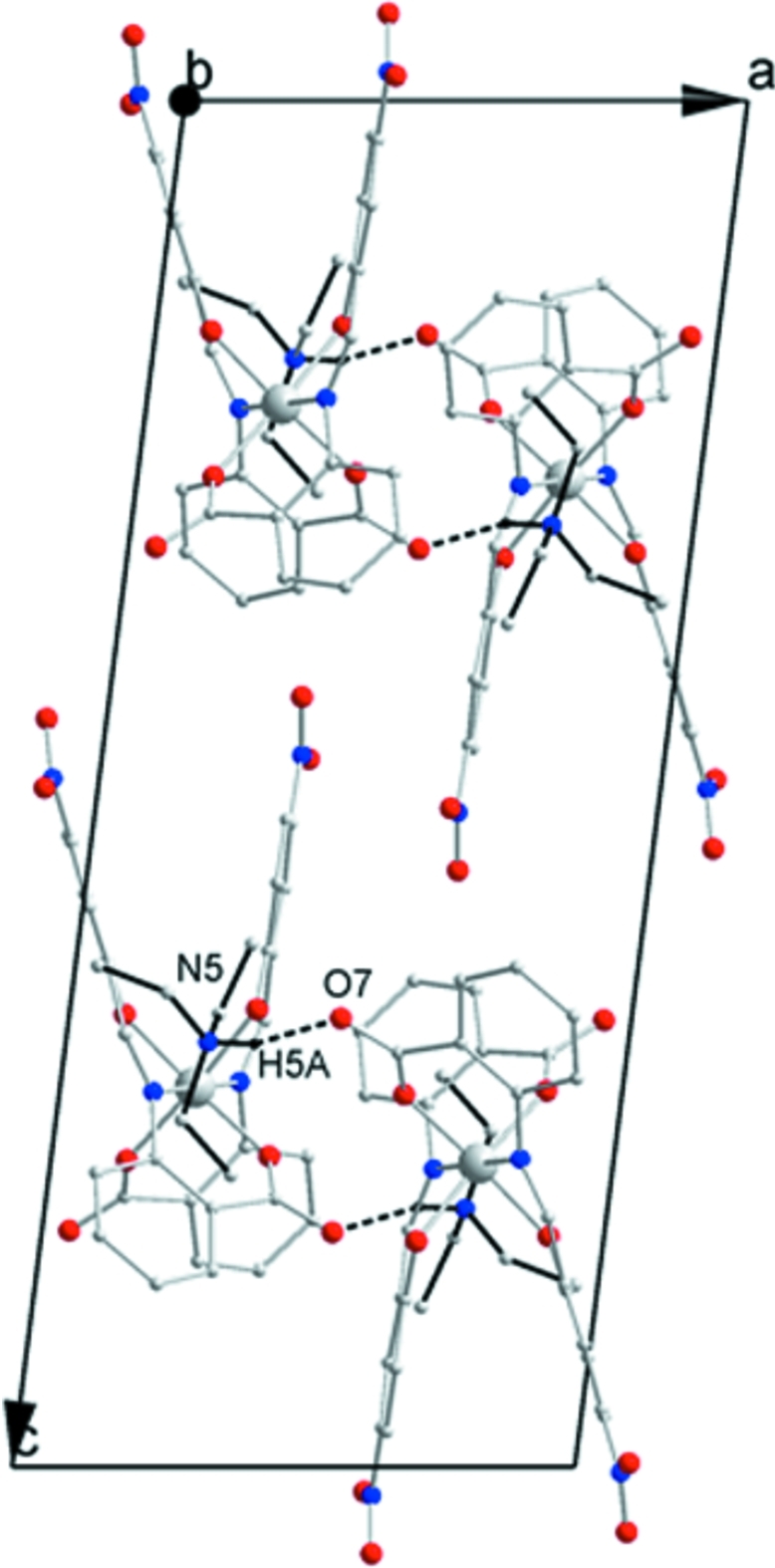
The packing of complex I, viewed down the b axis, showing N–H···O hydrogen bonds linking in pairs of the complex cations and anions. H atoms not involved in hydrogen bonding have been omitted.

**Fig. 3.**
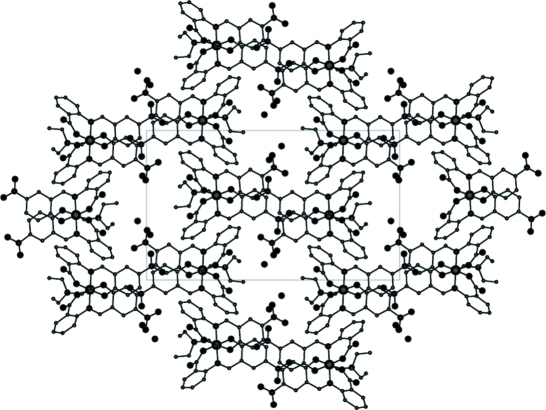
The packing of complex I, viewed down the a axis, demonstrating the voids occupied by water molecules.

**Fig. 4.**
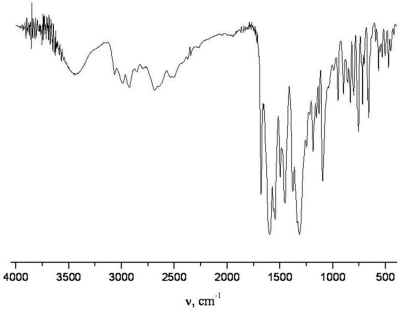
The IR spectrum of complex I (cm-1): 3440, 3065, 2989, 2928, 2861, 2793, 1678, 1592, 1550, 1508, 1457, 1371,1321, 1184, 1107, 955, 895, 862, 836, 801, 750, 715.

## supplementary crystallographic information

### Comment

There is growing interest in transition metal complexes with phenyl-salicyliden-imines (PSI) ligands due to their important applications and pharmacological activities (Rotondo *et al.*, 2009, Patel, 2009, Patel *et al.*, 2008, Laye & Sanudo, 2009). Here we represent a new complex with nitro-substituted PSI ligand, 2-(((2-hydroxy-5-nitrophenyl)methylene)amino)benzoic acid (H_2_L), wich was obtained as by-product during the invistigation of the system Co – FeCl_2_^.^4H_2_O – MnCl_2_^.^4H_2_O – H_2_L – Et_3_N – DMF.

The synthesis of {NH(C_2_H_5_)_3_}[Fe(C_14_H_8_NO_5_)_2_]^.^H_2_O, I, is carried out in air that leads to stabilization of iron(III) ion. The formation of the complex can be understood if one considers the following reaction scheme: FeCl_2_^.^4H_2_O + 2H_2_L + 3Et_3_N + 0.25O_2_→ (NHEt_3_)[Fe^III^*L*_2_] + 2Et_3_N^.^HCl + 9H_2_O

The crystal structure of I consists of triethylammonium cations and complex anions (Fig. 1) linked together by "strong" N—H···O hydrogen bonds (Fig. 2). The Fe centre is hexacoordinated in an axially elongated octahedral fashion (FeO_4_N_2_-chromophore) with oxidation state Fe(III), as it can be seen from close examination of the structured parameters and bond-valence sum calculations (Brown & Altermatt, 1985). Angular deviations from octahedral geometry are not significant, less than 5° for *cis-* and *trans-*angles.

In the crystal packing of I there are channels along the *a* axis (Fig. 3), accounting in total 610.7 Å^3^ per unit cell, *i.e.* some 16.2% of the total volume. The voids were examined using *PLATON* (Spek, 2009). The channels are occupied by disordered water molecules.

### Experimental

2-aminobenzoic acid (0.51 g, 3.75 mmol), 5-Nitrosalicylaldehyde (0.63 g, 3.75 mmol), and triethylamine (0.53 ml, 3.75 mmol) were dissolved in DMF (25 ml) in this order, forming a yellow solution and magnetically stirred at 50 – 60°C (10 min). Then, cobalt powder (0.08 g, 1.25 mmol), FeCl_2_^.^4H_2_O (0.25 g, 1.25 mmol) and MnCl_2_^.^4H_2_O (0.26 g, 1.25 mmol) were successfully added to the hot yellow solution with stirring about 2 h. Red crystals suitable for X-ray analysis were isolated by adding of *i*PrOH from the dark red solution after 1 day. Yield: 0.34 g, 0.32% (per Fe). Elemental analysis for C_34_H_34_Fe_1_N_5_O_11_ (Mr=744.52). Calcd: C, 54.85; N, 9.41; H, 4.6; Fe, 7.5. Found: C, 54.5; N, 9.6; H, 4.6; Fe, 7.2. The compound is sparingly soluble in DMSO and DMF, and it is stable in air. The infrared spectrum of solid recorded using KBr disk shows an adsorption at 3440 cm^-1^, which attributed to ν(OH) of H_2_O solvation molecules. The presence in IR spectrum of a band at 1680 cm^-1^ attributable to the ν(*C*=*N*)*_imine_* stretching frequency together with the absence of the band due to the ν(C=O) of the carbonyl indicates the formation of the Schiff base (Fig. 4).

### Refinement

Structure solution by direct methods in the space group *P2_1_/c*, followed by refinement, based on F^2^, of atomic coordinates and anisotropic displacement parameters, was performed using the programs *SHELX97* (Sheldrick, 2008) successively. H atoms bonded to C atoms were found in successive difference Fourier maps and refined using a riding model, with C–H = 0.93 Å and with *U*_iso_(H)=1.2*U*_eq_(C). O11 appeared to be highly disordered and was split in two positions with 0.4 and 0.6 occurrence. The H-atoms bonded to disordered oxygen O11 cannot be located from difference map. On this ground, both positions of O11 are refined without H atoms.

### Crystal data

**Table:**

(C_6_H_16_N)[Fe(C_14_H_8_N_2_O_5_)_2_]·H_2_O	*F*(000) = 1540
*M**_r_* = 742.50	*D*_x_ = 1.312 Mg m^−^^3^
Monoclinic, *P*2_1_/*c*	Mo *K*α radiation, λ = 0.71073 Å
Hall symbol: -P 2ybc	Cell parameters from 5575 reflections
*a* = 10.2688 (3) Å	θ = 5.8–25.0°
*b* = 14.7128 (4) Å	µ = 0.46 mm^−^^1^
*c* = 25.0800 (7) Å	*T* = 296 K
β = 97.230 (2)°	Prism, red
*V* = 3759.03 (18) Å^3^	0.35 × 0.05 × 0.04 mm
*Z* = 4	

### Data collection

**Table:**

Bruker APEXII CCD diffractometer	6432 independent reflections
Radiation source: fine-focus sealed tube	5305 reflections with *I* > 2σ(*I*)
graphite	*R*_int_ = 0.035
φ and ω scans	θ_max_ = 25.1°, θ_min_ = 5.8°
Absorption correction: multi-scan (*SADABS*; Bruker, 2005)	*h* = −12→12
*T*_min_ = 0.855, *T*_max_ = 0.982	*k* = −17→17
20469 measured reflections	*l* = −29→28

### Refinement

**Table:**

Refinement on *F*^2^	Primary atom site location: structure-invariant direct methods
Least-squares matrix: full	Secondary atom site location: difference Fourier map
*R*[*F*^2^ > 2σ(*F*^2^)] = 0.081	Hydrogen site location: inferred from neighbouring sites
*wR*(*F*^2^) = 0.242	H-atom parameters constrained
*S* = 1.13	*w* = 1/[σ^2^(*F*_o_^2^) + (0.1031*P*)^2^ + 8.9019*P*] where *P* = (*F*_o_^2^ + 2*F*_c_^2^)/3
6432 reflections	(Δ/σ)_max_ < 0.001
466 parameters	Δρ_max_ = 0.89 e Å^−^^3^
0 restraints	Δρ_min_ = −0.61 e Å^−^^3^

### Special details

**Table:**

Geometry. All e.s.d.'s (except the e.s.d. in the dihedral angle between two l.s. planes) are estimated using the full covariance matrix. The cell e.s.d.'s are taken into account individually in the estimation of e.s.d.'s in distances, angles and torsion angles; correlations between e.s.d.'s in cell parameters are only used when they are defined by crystal symmetry. An approximate (isotropic) treatment of cell e.s.d.'s is used for estimating e.s.d.'s involving l.s. planes.
Refinement. Refinement of *F*^2^ against ALL reflections. The weighted *R*-factor *wR* and goodness of fit *S* are based on *F*^2^, conventional *R*-factors *R* are based on *F*, with *F* set to zero for negative *F*^2^. The threshold expression of *F*^2^ > σ(*F*^2^) is used only for calculating *R*-factors(gt) *etc*. and is not relevant to the choice of reflections for refinement. *R*-factors based on *F*^2^ are statistically about twice as large as those based on *F*, and *R*- factors based on ALL data will be even larger.

### Fractional atomic coordinates and isotropic or equivalent isotropic displacement parameters (Å^2^)

**Table:**

	*x*	*y*	*z*	*U*_iso_*/*U*_eq_	Occ. (<1)
Fe1	0.76042 (6)	0.43361 (4)	0.27782 (3)	0.0393 (3)	
N1	0.6821 (4)	0.5564 (3)	0.28195 (17)	0.0488 (10)	
N2	0.6448 (5)	0.5933 (6)	0.5208 (2)	0.0771 (16)	
N3	0.8339 (4)	0.3095 (3)	0.27462 (17)	0.0495 (10)	
N4	1.0827 (7)	0.2486 (7)	0.5039 (3)	0.103 (2)	
N5	0.2528 (12)	0.4148 (8)	0.1890 (4)	0.139 (3)	
H5A	0.3355	0.3918	0.1905	0.167*	
O1	0.8649 (4)	0.4740 (3)	0.22465 (16)	0.0605 (10)	
O2	0.9514 (5)	0.5716 (3)	0.1731 (2)	0.0854 (14)	
O3	0.6678 (3)	0.3946 (2)	0.33491 (14)	0.0506 (8)	
O4	0.6579 (5)	0.5525 (5)	0.5635 (2)	0.0986 (18)	
O5	0.6314 (6)	0.6749 (5)	0.5180 (2)	0.1001 (18)	
O6	0.6131 (4)	0.4005 (3)	0.22741 (16)	0.0605 (10)	
O7	0.4835 (4)	0.3151 (4)	0.1717 (2)	0.0804 (13)	
O8	0.9015 (4)	0.4666 (3)	0.33112 (15)	0.0556 (9)	
O9	1.0951 (9)	0.1671 (6)	0.4971 (3)	0.137 (3)	
O10	1.1067 (8)	0.2867 (5)	0.5476 (2)	0.132 (3)	
O11A	0.741 (3)	0.1967 (14)	0.4845 (9)	0.160 (4)	0.40
O11B	0.6751 (18)	0.1050 (10)	0.5340 (6)	0.160 (4)	0.60
C1	0.8614 (6)	0.5491 (4)	0.1981 (2)	0.0588 (14)	
C2	0.7424 (6)	0.6079 (4)	0.1954 (2)	0.0593 (14)	
C3	0.6571 (6)	0.6104 (4)	0.2343 (2)	0.0556 (13)	
C4	0.6518 (5)	0.5896 (4)	0.3267 (2)	0.0509 (12)	
H4A	0.6265	0.6503	0.3269	0.061*	
C5	0.6544 (5)	0.5397 (4)	0.3761 (2)	0.0487 (11)	
C6	0.6607 (5)	0.4436 (4)	0.3777 (2)	0.0482 (11)	
C7	0.6597 (5)	0.3994 (5)	0.4274 (2)	0.0597 (14)	
H7A	0.6607	0.3362	0.4289	0.072*	
C8	0.6571 (6)	0.4482 (5)	0.4737 (2)	0.0673 (16)	
H8A	0.6607	0.4185	0.5066	0.081*	
C9	0.6491 (5)	0.5417 (5)	0.4714 (2)	0.0625 (15)	
C10	0.6460 (5)	0.5879 (4)	0.4237 (2)	0.0581 (14)	
H10A	0.6384	0.6509	0.4230	0.070*	
C11	0.5449 (8)	0.6639 (4)	0.2276 (3)	0.0780 (19)	
H11A	0.4867	0.6636	0.2532	0.094*	
C12	0.5208 (10)	0.7188 (5)	0.1812 (3)	0.096 (3)	
H12A	0.4460	0.7551	0.1763	0.115*	
C13	0.6048 (10)	0.7192 (5)	0.1442 (4)	0.099 (3)	
H13A	0.5885	0.7563	0.1140	0.118*	
C14	0.7160 (8)	0.6643 (5)	0.1507 (3)	0.0787 (19)	
H14A	0.7735	0.6652	0.1249	0.094*	
C15	0.5931 (5)	0.3311 (4)	0.1968 (2)	0.0573 (13)	
C16	0.7025 (6)	0.2670 (4)	0.1898 (2)	0.0597 (14)	
C17	0.8148 (6)	0.2571 (4)	0.2260 (2)	0.0554 (13)	
C18	0.8964 (5)	0.2712 (4)	0.3170 (2)	0.0531 (12)	
H18A	0.9137	0.2093	0.3152	0.064*	
C19	0.9408 (5)	0.3170 (4)	0.3664 (2)	0.0533 (13)	
C20	0.9450 (5)	0.4139 (4)	0.3703 (2)	0.0510 (12)	
C21	0.9988 (6)	0.4511 (5)	0.4194 (2)	0.0635 (15)	
H21A	1.0056	0.5140	0.4226	0.076*	
C22	1.0412 (6)	0.3991 (5)	0.4623 (3)	0.0700 (17)	
H22A	1.0744	0.4259	0.4948	0.084*	
C23	1.0348 (6)	0.3054 (6)	0.4577 (2)	0.0738 (18)	
C24	0.9887 (6)	0.2630 (5)	0.4103 (2)	0.0641 (15)	
H24A	0.9892	0.2000	0.4075	0.077*	
C25	0.9163 (7)	0.1966 (5)	0.2154 (3)	0.0730 (18)	
H25A	0.9941	0.1926	0.2386	0.088*	
C26	0.8961 (10)	0.1443 (6)	0.1697 (3)	0.104 (3)	
H26A	0.9595	0.1022	0.1628	0.125*	
C27	0.7844 (10)	0.1530 (6)	0.1342 (3)	0.107 (3)	
H27A	0.7737	0.1179	0.1031	0.129*	
C28	0.6886 (8)	0.2124 (5)	0.1438 (3)	0.086 (2)	
H28A	0.6128	0.2168	0.1194	0.104*	
C29	0.2660 (14)	0.5053 (12)	0.1683 (9)	0.165 (6)	
H29A	0.1813	0.5351	0.1667	0.198*	
H29B	0.3266	0.5386	0.1939	0.198*	
C30	0.309 (3)	0.5136 (18)	0.1172 (11)	0.274 (13)	
H30A	0.2453	0.5464	0.0935	0.411*	
H30B	0.3211	0.4542	0.1027	0.411*	
H30C	0.3914	0.5458	0.1207	0.411*	
C31	0.1695 (12)	0.3468 (13)	0.1528 (6)	0.172 (7)	
H31A	0.2154	0.3332	0.1222	0.206*	
H31B	0.1642	0.2907	0.1728	0.206*	
C32	0.0405 (15)	0.3743 (10)	0.1332 (6)	0.161 (5)	
H32A	0.0069	0.3363	0.1035	0.241*	
H32B	0.0415	0.4364	0.1216	0.241*	
H32C	−0.0145	0.3688	0.1613	0.241*	
C33	0.2234 (16)	0.4065 (15)	0.2459 (7)	0.192 (7)	
H33A	0.1400	0.4359	0.2488	0.230*	
H33B	0.2136	0.3426	0.2541	0.230*	
C34	0.323 (2)	0.4457 (12)	0.2862 (7)	0.204 (8)	
H34A	0.3114	0.4224	0.3211	0.306*	
H34B	0.3136	0.5107	0.2862	0.306*	
H34C	0.4086	0.4299	0.2778	0.306*	

### Atomic displacement parameters (Å^2^)

**Table:**

	*U*^11^	*U*^22^	*U*^33^	*U*^12^	*U*^13^	*U*^23^
Fe1	0.0418 (4)	0.0346 (4)	0.0414 (4)	0.0049 (3)	0.0053 (3)	−0.0007 (3)
N1	0.058 (2)	0.044 (2)	0.045 (2)	−0.0014 (19)	0.0095 (19)	−0.0020 (18)
N2	0.061 (3)	0.117 (5)	0.053 (3)	−0.003 (3)	0.008 (2)	−0.019 (3)
N3	0.053 (2)	0.051 (2)	0.043 (2)	0.0046 (19)	−0.0002 (18)	−0.0003 (18)
N4	0.104 (5)	0.128 (6)	0.070 (5)	−0.019 (5)	−0.013 (4)	0.038 (4)
N5	0.156 (9)	0.152 (9)	0.113 (7)	−0.003 (7)	0.033 (6)	−0.005 (6)
O1	0.056 (2)	0.073 (3)	0.054 (2)	0.0044 (19)	0.0148 (17)	−0.001 (2)
O2	0.084 (3)	0.093 (3)	0.086 (3)	−0.025 (3)	0.038 (3)	−0.003 (3)
O3	0.054 (2)	0.0494 (19)	0.049 (2)	−0.0039 (16)	0.0104 (15)	−0.0029 (16)
O4	0.084 (3)	0.165 (6)	0.047 (3)	0.004 (3)	0.009 (2)	−0.011 (3)
O5	0.124 (5)	0.112 (5)	0.068 (3)	0.006 (4)	0.026 (3)	−0.032 (3)
O6	0.055 (2)	0.067 (2)	0.058 (2)	0.0076 (18)	−0.0025 (17)	0.0048 (19)
O7	0.059 (2)	0.099 (3)	0.077 (3)	−0.004 (2)	−0.014 (2)	−0.010 (3)
O8	0.057 (2)	0.055 (2)	0.053 (2)	−0.0113 (17)	0.0005 (17)	0.0015 (17)
O9	0.182 (7)	0.120 (6)	0.098 (5)	0.012 (5)	−0.034 (5)	0.046 (4)
O10	0.166 (6)	0.170 (6)	0.049 (3)	−0.052 (5)	−0.028 (3)	0.030 (3)
O11A	0.213 (13)	0.127 (8)	0.131 (9)	0.005 (8)	−0.009 (8)	−0.001 (7)
O11B	0.213 (13)	0.127 (8)	0.131 (9)	0.005 (8)	−0.009 (8)	−0.001 (7)
C1	0.064 (3)	0.065 (3)	0.050 (3)	−0.015 (3)	0.014 (3)	−0.010 (3)
C2	0.075 (4)	0.052 (3)	0.051 (3)	−0.007 (3)	0.010 (3)	0.001 (2)
C3	0.074 (4)	0.043 (3)	0.051 (3)	0.004 (3)	0.009 (3)	0.000 (2)
C4	0.058 (3)	0.044 (3)	0.052 (3)	0.003 (2)	0.011 (2)	−0.005 (2)
C5	0.046 (3)	0.056 (3)	0.046 (3)	0.001 (2)	0.012 (2)	−0.006 (2)
C6	0.041 (2)	0.058 (3)	0.047 (3)	−0.002 (2)	0.008 (2)	−0.001 (2)
C7	0.056 (3)	0.070 (3)	0.055 (3)	0.002 (3)	0.015 (2)	0.010 (3)
C8	0.059 (3)	0.093 (5)	0.050 (3)	0.004 (3)	0.010 (3)	0.010 (3)
C9	0.050 (3)	0.096 (5)	0.043 (3)	0.000 (3)	0.009 (2)	−0.010 (3)
C10	0.048 (3)	0.069 (3)	0.058 (3)	0.003 (3)	0.010 (2)	−0.011 (3)
C11	0.106 (5)	0.064 (4)	0.064 (4)	0.027 (4)	0.014 (4)	0.007 (3)
C12	0.135 (7)	0.074 (5)	0.081 (5)	0.042 (5)	0.020 (5)	0.021 (4)
C13	0.146 (8)	0.071 (5)	0.079 (5)	0.023 (5)	0.018 (5)	0.024 (4)
C14	0.109 (5)	0.069 (4)	0.061 (4)	−0.002 (4)	0.023 (4)	0.008 (3)
C15	0.054 (3)	0.069 (4)	0.048 (3)	−0.001 (3)	0.003 (2)	0.007 (3)
C16	0.071 (3)	0.061 (3)	0.045 (3)	0.001 (3)	−0.004 (3)	−0.001 (2)
C17	0.066 (3)	0.052 (3)	0.048 (3)	0.008 (3)	0.007 (2)	−0.002 (2)
C18	0.058 (3)	0.049 (3)	0.051 (3)	0.008 (2)	0.001 (2)	0.001 (2)
C19	0.044 (3)	0.068 (3)	0.046 (3)	0.003 (2)	−0.002 (2)	0.002 (2)
C20	0.043 (3)	0.061 (3)	0.048 (3)	−0.007 (2)	0.001 (2)	0.002 (2)
C21	0.056 (3)	0.080 (4)	0.054 (3)	−0.011 (3)	0.003 (3)	−0.004 (3)
C22	0.065 (4)	0.092 (5)	0.052 (3)	−0.014 (3)	0.000 (3)	−0.004 (3)
C23	0.062 (4)	0.107 (5)	0.048 (3)	−0.008 (4)	−0.009 (3)	0.018 (3)
C24	0.057 (3)	0.076 (4)	0.058 (4)	0.006 (3)	−0.001 (3)	0.015 (3)
C25	0.082 (4)	0.075 (4)	0.061 (4)	0.030 (3)	0.006 (3)	−0.007 (3)
C26	0.141 (7)	0.093 (5)	0.073 (5)	0.051 (5)	−0.009 (5)	−0.026 (4)
C27	0.138 (7)	0.108 (6)	0.071 (5)	0.030 (6)	−0.007 (5)	−0.039 (5)
C28	0.097 (5)	0.091 (5)	0.065 (4)	0.013 (4)	−0.015 (4)	−0.019 (4)
C29	0.124 (9)	0.159 (13)	0.211 (19)	0.009 (9)	0.013 (10)	0.006 (12)
C30	0.38 (4)	0.26 (3)	0.19 (2)	−0.02 (2)	0.07 (2)	0.070 (18)
C31	0.092 (7)	0.285 (19)	0.132 (10)	−0.001 (10)	−0.013 (7)	−0.075 (11)
C32	0.165 (13)	0.163 (12)	0.159 (12)	−0.012 (10)	0.041 (10)	−0.060 (10)
C33	0.151 (12)	0.29 (2)	0.133 (12)	0.040 (13)	0.010 (9)	−0.069 (13)
C34	0.26 (2)	0.205 (16)	0.126 (11)	0.014 (14)	−0.043 (13)	−0.039 (11)

### Geometric parameters (Å, °)

**Table:**

Fe1—O3	1.904 (4)	C12—H12A	0.9300
Fe1—O8	1.906 (4)	C13—C14	1.391 (11)
Fe1—O1	1.908 (4)	C13—H13A	0.9300
Fe1—O6	1.909 (4)	C14—H14A	0.9300
Fe1—N3	1.981 (4)	C15—C16	1.493 (8)
Fe1—N1	1.985 (4)	C16—C17	1.382 (8)
N1—C4	1.299 (7)	C16—C28	1.399 (9)
N1—C3	1.431 (7)	C17—C25	1.420 (8)
N2—O5	1.209 (8)	C18—C19	1.432 (8)
N2—O4	1.219 (8)	C18—H18A	0.9300
N2—C9	1.460 (8)	C19—C24	1.396 (8)
N3—C18	1.300 (7)	C19—C20	1.429 (8)
N3—C17	1.436 (7)	C20—C21	1.397 (8)
N4—O9	1.220 (10)	C21—C22	1.348 (9)
N4—O10	1.230 (10)	C21—H21A	0.9300
N4—C23	1.462 (9)	C22—C23	1.385 (10)
N5—C29	1.443 (18)	C22—H22A	0.9300
N5—C33	1.50 (2)	C23—C24	1.373 (9)
N5—C31	1.537 (16)	C24—H24A	0.9300
N5—H5A	0.9100	C25—C26	1.375 (10)
O1—C1	1.289 (7)	C25—H25A	0.9300
O2—C1	1.225 (7)	C26—C27	1.367 (12)
O3—C6	1.301 (6)	C26—H26A	0.9300
O6—C15	1.280 (7)	C27—C28	1.359 (11)
O7—C15	1.241 (7)	C27—H27A	0.9300
O8—C20	1.288 (6)	C28—H28A	0.9300
C1—C2	1.492 (9)	C29—C30	1.41 (3)
C2—C3	1.392 (8)	C29—H29A	0.9700
C2—C14	1.394 (9)	C29—H29B	0.9700
C3—C11	1.387 (9)	C30—H30A	0.9600
C4—C5	1.436 (8)	C30—H30B	0.9600
C4—H4A	0.9300	C30—H30C	0.9600
C5—C10	1.400 (8)	C31—C32	1.413 (17)
C5—C6	1.417 (7)	C31—H31A	0.9700
C6—C7	1.409 (8)	C31—H31B	0.9700
C7—C8	1.368 (9)	C32—H32A	0.9600
C7—H7A	0.9300	C32—H32B	0.9600
C8—C9	1.379 (10)	C32—H32C	0.9600
C8—H8A	0.9300	C33—C34	1.46 (2)
C9—C10	1.373 (9)	C33—H33A	0.9700
C10—H10A	0.9300	C33—H33B	0.9700
C11—C12	1.412 (10)	C34—H34A	0.9600
C11—H11A	0.9300	C34—H34B	0.9600
C12—C13	1.346 (12)	C34—H34C	0.9600
			
O3—Fe1—O8	87.53 (16)	O7—C15—O6	121.5 (6)
O3—Fe1—O1	175.55 (16)	O7—C15—C16	118.0 (6)
O8—Fe1—O1	88.07 (17)	O6—C15—C16	120.6 (5)
O3—Fe1—O6	89.54 (16)	C17—C16—C28	118.1 (6)
O8—Fe1—O6	176.91 (17)	C17—C16—C15	124.6 (5)
O1—Fe1—O6	94.87 (18)	C28—C16—C15	117.3 (5)
O3—Fe1—N3	88.95 (17)	C16—C17—C25	121.0 (5)
O8—Fe1—N3	90.20 (17)	C16—C17—N3	120.9 (5)
O1—Fe1—N3	90.46 (18)	C25—C17—N3	118.1 (5)
O6—Fe1—N3	90.70 (17)	N3—C18—C19	125.1 (5)
O3—Fe1—N1	89.48 (17)	N3—C18—H18A	117.5
O8—Fe1—N1	90.51 (17)	C19—C18—H18A	117.5
O1—Fe1—N1	91.17 (18)	C24—C19—C20	120.6 (5)
O6—Fe1—N1	88.50 (18)	C24—C19—C18	117.1 (5)
N3—Fe1—N1	178.24 (18)	C20—C19—C18	122.1 (5)
C4—N1—C3	118.4 (4)	O8—C20—C21	119.8 (5)
C4—N1—Fe1	122.0 (4)	O8—C20—C19	123.0 (5)
C3—N1—Fe1	119.6 (3)	C21—C20—C19	117.2 (5)
O5—N2—O4	122.6 (6)	C22—C21—C20	122.2 (6)
O5—N2—C9	118.8 (6)	C22—C21—H21A	118.9
O4—N2—C9	118.5 (7)	C20—C21—H21A	118.9
C18—N3—C17	117.6 (4)	C21—C22—C23	119.4 (6)
C18—N3—Fe1	121.1 (4)	C21—C22—H22A	120.3
C17—N3—Fe1	121.2 (3)	C23—C22—H22A	120.3
O9—N4—O10	124.1 (7)	C24—C23—C22	122.2 (6)
O9—N4—C23	118.8 (8)	C24—C23—N4	118.1 (7)
O10—N4—C23	117.1 (9)	C22—C23—N4	119.6 (7)
C29—N5—C33	117.3 (14)	C23—C24—C19	118.2 (6)
C29—N5—C31	117.2 (13)	C23—C24—H24A	120.9
C33—N5—C31	110.2 (12)	C19—C24—H24A	120.9
C29—N5—H5A	103.2	C26—C25—C17	118.0 (6)
C33—N5—H5A	103.2	C26—C25—H25A	121.0
C31—N5—H5A	103.2	C17—C25—H25A	121.0
C1—O1—Fe1	130.3 (4)	C27—C26—C25	121.1 (7)
C6—O3—Fe1	122.5 (3)	C27—C26—H26A	119.4
C15—O6—Fe1	130.9 (4)	C25—C26—H26A	119.4
C20—O8—Fe1	122.8 (3)	C28—C27—C26	120.8 (7)
O2—C1—O1	121.5 (6)	C28—C27—H27A	119.6
O2—C1—C2	119.1 (6)	C26—C27—H27A	119.6
O1—C1—C2	119.3 (5)	C27—C28—C16	120.9 (7)
C3—C2—C14	118.2 (6)	C27—C28—H28A	119.5
C3—C2—C1	124.7 (5)	C16—C28—H28A	119.5
C14—C2—C1	117.2 (6)	C30—C29—N5	117.5 (17)
C11—C3—C2	121.0 (5)	C30—C29—H29A	107.9
C11—C3—N1	118.4 (5)	N5—C29—H29A	107.9
C2—C3—N1	120.5 (5)	C30—C29—H29B	107.9
N1—C4—C5	124.9 (5)	N5—C29—H29B	107.9
N1—C4—H4A	117.5	H29A—C29—H29B	107.2
C5—C4—H4A	117.5	C29—C30—H30A	109.5
C10—C5—C6	119.3 (5)	C29—C30—H30B	109.5
C10—C5—C4	118.6 (5)	H30A—C30—H30B	109.5
C6—C5—C4	122.0 (5)	C29—C30—H30C	109.5
O3—C6—C7	118.8 (5)	H30A—C30—H30C	109.5
O3—C6—C5	122.5 (5)	H30B—C30—H30C	109.5
C7—C6—C5	118.7 (5)	C32—C31—N5	116.5 (13)
C8—C7—C6	120.8 (6)	C32—C31—H31A	108.2
C8—C7—H7A	119.6	N5—C31—H31A	108.2
C6—C7—H7A	119.6	C32—C31—H31B	108.2
C7—C8—C9	119.6 (6)	N5—C31—H31B	108.2
C7—C8—H8A	120.2	H31A—C31—H31B	107.3
C9—C8—H8A	120.2	C31—C32—H32A	109.5
C10—C9—C8	121.8 (6)	C31—C32—H32B	109.5
C10—C9—N2	118.9 (6)	H32A—C32—H32B	109.5
C8—C9—N2	119.3 (6)	C31—C32—H32C	109.5
C9—C10—C5	119.6 (6)	H32A—C32—H32C	109.5
C9—C10—H10A	120.2	H32B—C32—H32C	109.5
C5—C10—H10A	120.2	C34—C33—N5	114.7 (16)
C3—C11—C12	118.8 (7)	C34—C33—H33A	108.6
C3—C11—H11A	120.6	N5—C33—H33A	108.6
C12—C11—H11A	120.6	C34—C33—H33B	108.6
C13—C12—C11	120.6 (7)	N5—C33—H33B	108.6
C13—C12—H12A	119.7	H33A—C33—H33B	107.6
C11—C12—H12A	119.7	C33—C34—H34A	109.5
C12—C13—C14	120.3 (7)	C33—C34—H34B	109.5
C12—C13—H13A	119.9	H34A—C34—H34B	109.5
C14—C13—H13A	119.9	C33—C34—H34C	109.5
C13—C14—C2	121.0 (7)	H34A—C34—H34C	109.5
C13—C14—H14A	119.5	H34B—C34—H34C	109.5
C2—C14—H14A	119.5		
			
O3—Fe1—N1—C4	30.7 (4)	C7—C8—C9—C10	−1.2 (9)
O8—Fe1—N1—C4	−56.8 (4)	C7—C8—C9—N2	179.2 (5)
O1—Fe1—N1—C4	−144.9 (4)	O5—N2—C9—C10	4.0 (9)
O6—Fe1—N1—C4	120.2 (4)	O4—N2—C9—C10	−174.4 (6)
N3—Fe1—N1—C4	57 (6)	O5—N2—C9—C8	−176.5 (6)
O3—Fe1—N1—C3	−149.0 (4)	O4—N2—C9—C8	5.2 (8)
O8—Fe1—N1—C3	123.5 (4)	C8—C9—C10—C5	−1.6 (8)
O1—Fe1—N1—C3	35.4 (4)	N2—C9—C10—C5	177.9 (5)
O6—Fe1—N1—C3	−59.4 (4)	C6—C5—C10—C9	2.5 (8)
N3—Fe1—N1—C3	−123 (6)	C4—C5—C10—C9	−179.6 (5)
O3—Fe1—N3—C18	−54.7 (4)	C2—C3—C11—C12	−2.4 (10)
O8—Fe1—N3—C18	32.8 (4)	N1—C3—C11—C12	178.8 (6)
O1—Fe1—N3—C18	120.9 (4)	C3—C11—C12—C13	0.2 (13)
O6—Fe1—N3—C18	−144.2 (4)	C11—C12—C13—C14	0.9 (14)
N1—Fe1—N3—C18	−81 (6)	C12—C13—C14—C2	0.2 (13)
O3—Fe1—N3—C17	122.5 (4)	C3—C2—C14—C13	−2.3 (10)
O8—Fe1—N3—C17	−150.0 (4)	C1—C2—C14—C13	178.0 (7)
O1—Fe1—N3—C17	−61.9 (4)	Fe1—O6—C15—O7	170.2 (4)
O6—Fe1—N3—C17	32.9 (4)	Fe1—O6—C15—C16	−10.4 (8)
N1—Fe1—N3—C17	96 (6)	O7—C15—C16—C17	−158.3 (6)
O3—Fe1—O1—C1	−108 (2)	O6—C15—C16—C17	22.3 (9)
O8—Fe1—O1—C1	−100.2 (5)	O7—C15—C16—C28	20.7 (9)
O6—Fe1—O1—C1	78.8 (5)	O6—C15—C16—C28	−158.7 (6)
N3—Fe1—O1—C1	169.6 (5)	C28—C16—C17—C25	3.2 (10)
N1—Fe1—O1—C1	−9.8 (5)	C15—C16—C17—C25	−177.9 (6)
O8—Fe1—O3—C6	46.6 (4)	C28—C16—C17—N3	−178.6 (6)
O1—Fe1—O3—C6	54 (2)	C15—C16—C17—N3	0.4 (9)
O6—Fe1—O3—C6	−132.5 (4)	C18—N3—C17—C16	146.0 (6)
N3—Fe1—O3—C6	136.8 (4)	Fe1—N3—C17—C16	−31.3 (7)
N1—Fe1—O3—C6	−44.0 (4)	C18—N3—C17—C25	−35.7 (8)
O3—Fe1—O6—C15	−102.1 (5)	Fe1—N3—C17—C25	147.0 (5)
O8—Fe1—O6—C15	−120 (3)	C17—N3—C18—C19	170.4 (5)
O1—Fe1—O6—C15	77.3 (5)	Fe1—N3—C18—C19	−12.3 (8)
N3—Fe1—O6—C15	−13.2 (5)	N3—C18—C19—C24	170.6 (6)
N1—Fe1—O6—C15	168.4 (5)	N3—C18—C19—C20	−13.5 (9)
O3—Fe1—O8—C20	46.8 (4)	Fe1—O8—C20—C21	−149.8 (4)
O1—Fe1—O8—C20	−132.6 (4)	Fe1—O8—C20—C19	30.0 (7)
O6—Fe1—O8—C20	65 (3)	C24—C19—C20—O8	−179.6 (5)
N3—Fe1—O8—C20	−42.1 (4)	C18—C19—C20—O8	4.6 (8)
N1—Fe1—O8—C20	136.3 (4)	C24—C19—C20—C21	0.2 (8)
Fe1—O1—C1—O2	165.6 (4)	C18—C19—C20—C21	−175.5 (5)
Fe1—O1—C1—C2	−17.5 (8)	O8—C20—C21—C22	177.5 (6)
O2—C1—C2—C3	−155.9 (6)	C19—C20—C21—C22	−2.4 (9)
O1—C1—C2—C3	27.0 (8)	C20—C21—C22—C23	1.7 (10)
O2—C1—C2—C14	23.7 (8)	C21—C22—C23—C24	1.1 (11)
O1—C1—C2—C14	−153.3 (6)	C21—C22—C23—N4	179.0 (6)
C14—C2—C3—C11	3.4 (9)	O9—N4—C23—C24	9.0 (12)
C1—C2—C3—C11	−176.9 (6)	O10—N4—C23—C24	−170.3 (7)
C14—C2—C3—N1	−177.8 (5)	O9—N4—C23—C22	−169.0 (8)
C1—C2—C3—N1	1.9 (8)	O10—N4—C23—C22	11.7 (11)
C4—N1—C3—C11	−36.8 (8)	C22—C23—C24—C19	−3.2 (10)
Fe1—N1—C3—C11	142.9 (5)	N4—C23—C24—C19	178.9 (6)
C4—N1—C3—C2	144.4 (5)	C20—C19—C24—C23	2.5 (9)
Fe1—N1—C3—C2	−35.9 (7)	C18—C19—C24—C23	178.4 (6)
C3—N1—C4—C5	171.3 (5)	C16—C17—C25—C26	−3.8 (11)
Fe1—N1—C4—C5	−8.4 (7)	N3—C17—C25—C26	177.9 (7)
N1—C4—C5—C10	166.9 (5)	C17—C25—C26—C27	3.0 (14)
N1—C4—C5—C6	−15.2 (8)	C25—C26—C27—C28	−1.5 (17)
Fe1—O3—C6—C7	−145.1 (4)	C26—C27—C28—C16	0.8 (15)
Fe1—O3—C6—C5	34.6 (6)	C17—C16—C28—C27	−1.6 (12)
C10—C5—C6—O3	179.6 (5)	C15—C16—C28—C27	179.3 (8)
C4—C5—C6—O3	1.8 (8)	C33—N5—C29—C30	172.4 (18)
C10—C5—C6—C7	−0.6 (7)	C31—N5—C29—C30	−53 (2)
C4—C5—C6—C7	−178.5 (5)	C29—N5—C31—C32	−53.9 (18)
O3—C6—C7—C8	177.6 (5)	C33—N5—C31—C32	83.7 (17)
C5—C6—C7—C8	−2.2 (8)	C29—N5—C33—C34	−60.7 (19)
C6—C7—C8—C9	3.1 (9)	C31—N5—C33—C34	161.8 (15)

### Hydrogen-bond geometry (Å, °)

**Table:**

*D*—H···*A*	*D*—H	H···*A*	*D*···*A*	*D*—H···*A*
N5—H5A···O7	0.91	2.00	2.865 (13)	159

## References

[bb1] Brown, I. D. & Altermatt, D. (1985). *Acta Cryst.* B**41**, 244–247.

[bb2] Bruker (2005). *APEX2*, *SAINT* and *SADABS* Bruker AXS Inc., Madison, Wisconsin, USA.

[bb3] Laye, R. & Sanudo, E. C. (2009). *Inorg. Chim. Acta*, **362**, 2205–2212.

[bb4] Lu, L.-P., Yao, S.-Q. & Zhu, M.-L. (2006). *Acta Cryst.* C**62**, m220–m222. 10.1107/S0108270106013242 16679589

[bb5] Patel, R. N. (2009). *Indian J. Chem.* A**48**, 1370–1377.

[bb6] Patel, R. N., Gundla, V. L. N. & Patel, D. K. (2008). *Polyhedron*, **27**, 1054–1060.

[bb7] Rosair, G. M., Dey, D. K., Samanta, B. & Mitra, S. (2002). *Acta Cryst.* C**58**, m266–m267. 10.1107/s0108270102003530 11932539

[bb8] Rotondo, A., Bruno, G., Brancatelli, G., Nicolo, F. & Armentano, D. (2009). *Inorg. Chim. Acta*, **326**, 247–252.

[bb9] Sheldrick, G. M. (2008). *Acta Cryst.* A**64**, 112–122. 10.1107/S0108767307043930 18156677

[bb10] Spek, A. L. (2009). *Acta Cryst.* D**65**, 148–155. 10.1107/S090744490804362X PMC2631630 19171970

[bb11] Westrip, S. P. (2010). *J. Appl. Cryst.* **43**, 920–925.

